# Assessment of the AquaCrop Model for Use in Simulation of Irrigated Winter Wheat Canopy Cover, Biomass, and Grain Yield in the North China Plain

**DOI:** 10.1371/journal.pone.0086938

**Published:** 2014-01-28

**Authors:** Xiu-liang Jin, Hai-kuan Feng, Xin-kai Zhu, Zhen-hai Li, Sen-nan Song, Xiao-yu Song, Gui-jun Yang, Xin-gang Xu, Wen-shan Guo

**Affiliations:** 1 Key Laboratory of Crop Genetics and Physiology of Jiangsu Province, Yangzhou University, Yangzhou, China; 2 Beijing Research Center for Information Technology in Agriculture, Beijing Academy of Agriculture and Forestry Sciences, Beijing, China; 3 National Engineering Research Center for Information Technology in Agriculture, Beijing, China; Tennessee State University, United States of America

## Abstract

Improving winter wheat water use efficiency in the North China Plain (NCP), China is essential in light of current irrigation water shortages. In this study, the AquaCrop model was used to calibrate, and validate winter wheat crop performance under various planting dates and irrigation application rates. All experiments were conducted at the Xiaotangshan experimental site in Beijing, China, during seasons of 2008/2009, 2009/2010, 2010/2011 and 2011/2012. This model was first calibrated using data from 2008/2009 and 2009/2010, and subsequently validated using data from 2010/2011 and 2011/2012. The results showed that the simulated canopy cover (CC), biomass yield (BY) and grain yield (GY) were consistent with the measured CC, BY and GY, with corresponding coefficients of determination (R^2^) of 0.93, 0.91 and 0.93, respectively. In addition, relationships between BY, GY and transpiration (T), (R^2^ = 0.57 and 0.71, respectively) was observed. These results suggest that frequent irrigation with a small amount of water significantly improved BY and GY. Collectively, these results indicate that the AquaCrop model can be used in the evaluation of various winter wheat irrigation strategies. The AquaCrop model predicted winter wheat CC, BY and GY with acceptable accuracy. Therefore, we concluded that AquaCrop is a useful decision-making tool for use in efforts to optimize wheat winter planting dates, and irrigation strategies.

## Introduction

Winter wheat (*Triticum aestivum* L.) is an important staple food crop for the majority of the North China Plain (NCP) population [1]. However, increasing industrial, and domestic water use has resulted in a reduction in water available for irrigation of these crops. Thus there is a growing need for improvement to this region’s agriculture water resources management, especially given increasing food demands of the region’s increasing population.

It is widely known that well-timed irrigation can substantially increase water use efficiency (WUE) [2], [3], providing an optimal growth environment throughout the season [4], [5]. In fact, various studies have described several such irrigation strategies for use by farmers [6]–[16]. Since the mid-1960s, the relationship between water and crop yield has been described with both empirical and mechanistic models [17]–[20]. For example, De Wit [21] proposed that a linear relationship between yield and water consumption exists. In contrast, Downey [22], via deficit irrigation studies, suggested that there exists a nonlinear relationship between water and yield. Based on the above studies, the Minhas model [23], Rao model [18], Blank model [24], and the Stewart model [25] were developed. More recently, Wang and Sun [26] showed that a quadratic relationship between crop yield and crop water consumption did in fact exist. Their work was followed by Kang et al. [27] in which a multiple and synergistic model (developed under deficit irrigation conditions) was proposed. At present, the simulation of the soil-plant-atmosphere continuum remains an important part of such research, especially with regard to expansion of the application range of resulting models to a wider array of cropping systems.

Therefore, the Food and Agricultural Organization (FAO) developed the AquaCrop model in an effort to meet this need in 2009. This model was originated from the “yield response to water” data of Doorenbos and Kassam [28], and evolved to a normalized crop water productivity (NCWP) concept [29]. Compared with other models, AquaCrop is relatively simple to operate by those with little, or no research experience, and allows for simulation of crop performance in multiple scenarios. In addition to a high level of accuracy, this robust model requires a limited set of input parameters, most of which are relatively easy to acquire [29], [30]. The AquaCrop model is also capable of predicting crop productivity, water requirements, and water use efficiency under water-limiting conditions [31]. To date, this model has been successfully tested for cotton [32], [33], maize [30], [34]–[38], wheat [39]–[42], sugar beet [37], sunflower [37], [43], groundnut [44], potato [45], [46], quinoa [16], Teff [47], barley [48], [49], green onion [50] and tomato [51] under a wide-range of environments. Previous studies have demonstrated that the AquaCrop model accurately simulates crop canopy cover (CC), biomass yield (BY) and grain yield (GY) under both regular, and deficit irrigation, and in low soil fertility conditions. In such unreliable water-limited environments as the NCP, the AquaCrop model is a potentially valuable tool for use in efforts to maximize this region’s winter wheat yield. Therefore, the objective of this study was to validate this model in simulating the effects of planting date, and multiple irrigation scenarios on: (1) canopy cover, (2) biomass yield, (3) grain yield, and (4) water use efficiency of winter wheat in the NCP. These data will provide some guidelines for efforts to optimize irrigation management for winter wheat crops in this region.

## Materials and Methods

### Study Site

This field experiments were conducted in the 2008/2009, 2009/2010, 2010/2011 and 2011/2012 growing seasons at the Xiaotangshan experimental site (44.17° N, 116.433°E), Beijing, PR China. This area is representative of the overall soil and crop management practices in this region. The soil is fine-loamy, with a nitrate Nitrogen (NO_3_–N) content of 3.16–14.82 mg kg^−1^, an ammonium Nitrogen (NH_3_–N) content of 10.20–12.32 mg kg^−1^, an Olsen P of 3.14–21.18 mg kg^−1^, an exchangeable K of 86.83–120.62 mg kg^−1^, and an organic matter content of 15.84–20.24g kg^−1^ with in the uppermost 0–30 cm layer. Beijing is characterized by a typical continental climate, with maximum temperatures of 26.1°C in the summer and minimum temperatures of −4.7°C in the winter. Throughout all seasons, the temperature fluctuated daily with significant differences between night and day. During this experimental period, the average annual precipitation was 650 mm, and the frost-free period was on average 180 days.

Local winter wheat cultivars and planting dates are shown in Table 1. Each plot area is 100 m^2^ in 2008, 2009 and 2010, and 300 m^2^ in 2011. The experiment was designed as a 2-way factorial arrangement of treatments in a randomized complete block design, with three replications for each treatment. Plot management followed local standard practices (weed control, pest management and fertilizer application) for wheat production in this region. The Xiaotangshan Experimental Site belongs to the National Engineering Research Center for Information Technology in Agriculture. It gives some permission for us to study relative agriculture research within this area. We confirm that the field studies did not involve endangered or protected species.

**Table 1:** Winter wheat cultivars and planting dates were selected in the 2008, 2009, 2010 and 2011.

Winter wheat cultivars	Planting dates
Nongda195, Jingdong8, Jing9428	Sep. 28th, Oct. 7th, and Oct. 20th, 2008
Nongda195, Jingdong13, Jing9428	Sep. 25th, Oct. 5th, and Oct. 15th, 2009
Nongda195, Yannong19, Jing9428	Sep. 25th, Oct. 5th, and Oct. 15th, 2010
Nongda211, Zhongmai175, Jingdong8, Jing9843	Sep. 25th, 2011

Note: There are three winter wheat cultivars and each has three planting dates in 2008, 2009 and 2010. In 2011, four cultivars are planted on the same date.

### Climate Data Collection and Analysis

**Figure 1:**
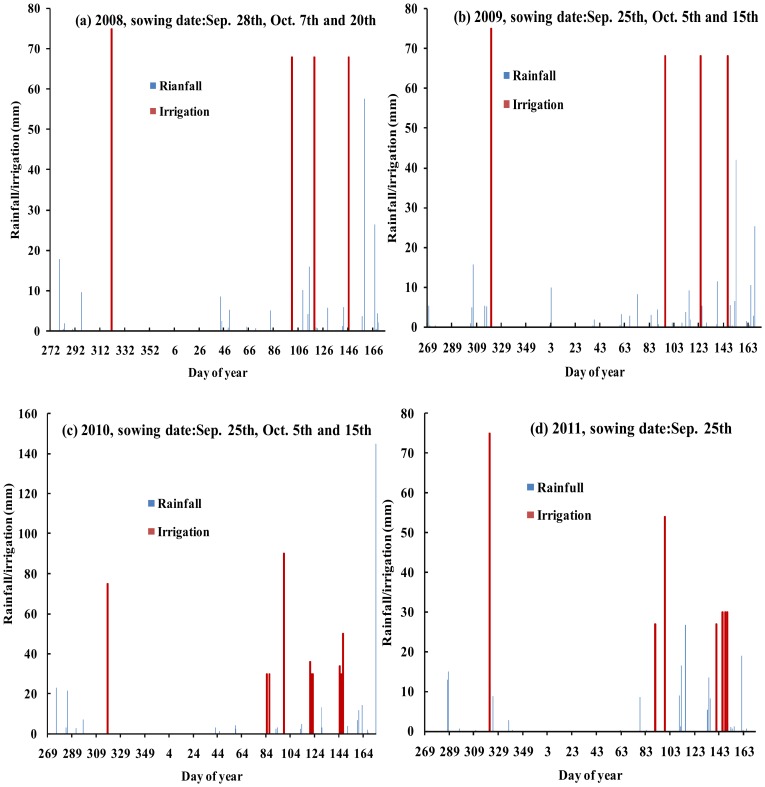
Daily rainfall, and supplemental irrigation for the Xiaotangshan site during the cropping seasons 2008/2009 (a), 2009/2010(b), 2010/2011 (c) and 2011/2012(d).

Climate data for the experimental site was obtained from the local Xiaotangshan meteorological station. The daily reference evapotranspiration (ET_o_) for the growing season from 2008 to 2012 was calculated based on the FAO Penman-Monteith method as described in Allen et al. [52], and the ET_o_ calculator (FAO, 2009) [53]. Daily maximum and minimum temperature, relative humidity, wind speed, rainfall, and total sunshine hours were recorded directly at the Xiaotangshan experimental site. The total rainfall, from sowing to harvest was 199, 208, 145 and 168 mm in 2008/2009, 2009/2010, 2010/2011 and 2011/2012, respectively. Supplemental irrigation was applied to treatments following cessation of any rain events (Fig. 1a–d and Table 2).

**Table 2:** Irrigation schedule during experimental period (2008/2009, 2009/2010, 2010/2011 and 2011/2012).

Day ofyear	Year	Month	Day	Irrigationamount (mm)
321	2008	11	16	75
100	2009	4	10	68
118	2009	4	28	68
146	2009	5	26	68
320	2009	11	15	75
95	2010	4	5	68
124	2010	5	4	68
146	2010	5	26	68
318	2010	11	13	75
84	2011	3	24	30
86	2011	3	26	30
98	2011	4	7	90
120	2011	4	29	36
121	2011	4	30	30
122	2011	5	2	30
144	2011	5	23	34
145	2011	5	24	30
146	2011	5	25	30
147	2011	5	26	54
321	2011	11	16	75
90	2012	3	30	27
98	2012	4	7	54
140	2012	5	19	27
145	2012	5	24	30
147	2012	5	26	30
149	2012	5	28	30

### Soil Data of the Experimental Site

The soil at the Xiaotangshan experimental site represents the major soil type (fine-loamy) on which winter wheat is grown in NCP. The soil was at maximum field capacity (27.3% at 0.0–0.1 m, 27.3% at 0.1–0.2 m and 34.8% at 0.2–0.3 m) during sowing and early establishment. The physical soil characteristics were measured directly in the field and used for input into AquaCrop (Table 3).

**Table 3:** Physical soil characteristics of the Xiaotangshan experimental site.

Site	Soil texture	Groundwater table	Depth (m)	Moisture content (vol%)			Ksat (mm day^−1^)	CN
				Sat	FC	WP		
Xiaotangshan	fine-loamy	3.5 m	0.0–0.1	51.1	27.3	8.8	240	75
			0.1–0.2	51.3	27.3	8.7	240	
			0.2–0.3	54.7	34.8	13.2	224	

Note: FC, field capacity; WP, wilting point; Sat, water content at saturation; CN, curve number; and K_sat_, saturation hydraulic conductivity describes water movement through saturated media. The values of saturated hydraulic conductivity in soils vary within a wide range of several orders of magnitude, depending on the soil material.

### Field Experiments and Crop Data Collection

In 2008/2009, 2009/2010, 2010/2011, and 2011/2012 aboveground biomass was determined 5–6 times from a 0.25 m^2^ area by randomly cutting four representative plants from each plot. All plant samples were heated to 105°C, oven dried at 70°C to a constant weight, and final dry weight (DW) recorded.

Leaf area index (LAI) was estimated using the following two methods:

1. By multiplying the plant population by the leaf area per plant as described in Kar et al. [54]. Area of the leaf was measured manually from 20 plants using a straightedge. Counting of plant populations was conducted manually from a 0.1 m^2^ area. The LAI equation is as follows:
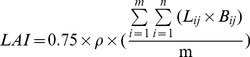
(1)Where ρ is plant density, *m* is the number of measured plants, *L_ij_* is leaf length, *B_ij_* is the maximum leaf width, and *n* is the number of leaves of the nth plant.
2. The LAI-2000 Plant Canopy Analyzer (LI-COR Inc., Lincoln, NE, USA) was used in measuring for determination of LAI. The resulting values were similar to those obtained via the manual LAI calculations, thus the LAI-2000 data was used as the model input data.

Canopy cover was estimated from different irrigation treatments in 2008/2009, 2009/2010, 2010/2011, and 2011/2012 based on Hsiao et al. [30] using the following:
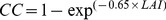
(2)where *CC* is canopy cover, and *LAI* is the leaf area index.

Grain yield was measured following maturation from samples obtained from a 1.5 m^2^ area in each plot, with three replications for each treatment. Collected grain was dried and weighed on an electronic scale (±0.01 g). As there were no significant differences between winter wheat varieties in many of the measured characteristics (e.g. phenological development, canopy cover, etc.), the average grain yield of the different varieties was considered in model simulations.

### Water Use Efficiency (WUE)

Water use efficiency (WUE) is defined as the grain yield per unit amount of water consumed [55]. In this study grain water use efficiency (Grain-WUE), and biomass water use efficiency (Biomass-WUE) were calculated using Eq. (3) and Eq. (4), respectively, as in Araya et al. (2010b) [48]:
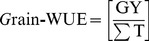
(3)

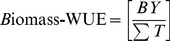
(4)Where *GY* is the grain yield kg ha^−1^ (measured), *T* is the transpiration as determined using AquaCrop model, and *BY* is the total final aboveground biomass yield in kg ha^−1^ (measured).

### Description of AquaCrop Model

The AquaCrop model was proposed by the FAO in 2009, with a detailed description presented in Steduto et al. [29], and Raes et al. [31]. The model computes a daily water balance, and separates evapotranspiration into evaporation and transpiration components. Transpiration is correlated with canopy cover, which is proportional to the degree of soil cover, and evaporation is proportional to the area of soil not covered by vegetation. The crop’s stomata conductance, canopy senescence, leaf growth, and yield response to water stress are modeled using four stress coefficients (stomata closure, leaf expansion, canopy senescence, and change in harvest index (HI)). The model subsequently estimates yield from the daily crop transpiration values.

In general, the normalized crop water productivity (NCWP) is considered constant for a given climate condition and crop (For crops not nutrient-limited, the model provides categories ranging from slight to severe deficiencies corresponding to lower water productivity (WP).) is applicable for using in different locations, seasons, and even future climates [29]. Depending on the crop, NCWP increases slightly with an increase in atmospheric CO_2_ concentration [29]. NCWP is set between 13 and 20 g m^−2^ for C_3_ crops. For example, NCWP is set at 15 g m^−2^ for the winter wheat according to the AquaCrop Manual (Annex I Section I.10 Wheat, Pages A39–A42) [56], [57]. In our current study, we have not included any of the water stress study data; therefore NCWP remained at 15 g m^−2^ for the winter wheat. The crop’s daily aboveground biomass is calculated using NCWP from the AquaCrop model [29], [30]. Biomass yield (BY) is calculated by multiplying NCWP by the ratio of crop transpiration (T), and evapotranspiration (ET_o_), following calculation of BY (its harvestable portion), and the grain yield (GY) is determined via harvest index (HI).

These changes are described by the following Eqs. (5) and (6):
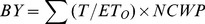
(5)

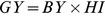
(6)Where *BY* is biomass yield in kg ha^−1^, *T* is crop transpiration in mm, *ET_o_* is evapotranspiration in mm, *NCWP* is the normalized crop water productivity in g m^−2^, *HI* is harvest index, and *GY* is grain yield in kg ha^−1^.

### Data Analysis

Winter wheat canopy cover (CC), biomass yield (BY) and grain yield (GY) in AquaCrop were calibrated using the measured data sets of 2008/2009 and 2009/2010, and validated using the 2010/2011 and 2011/2012 measured data sets. The good fit regression equation between the simulated and observed values was corroborated using prediction error statistics. The coefficient of determination (R^2^), root mean square error (RMSE), and model efficiency (E) were used as the error statistics to evaluate both calibration and validation results. The E and R^2^ were used to access the predictive power of the model, and the RMSE indicated the error in model prediction. In this study, the prediction model output for CC, GY and BY during harvest was used for model evaluation. These statistical indices were used to compare measured and simulated values. Model performance was assessed using E (Nash and Sutcliffe, 1970) as follows:
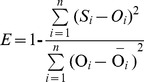
(7)where *S_i_* and *O_i_* are predicted, and observed data, respectively. 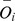 is the mean value of *O_i_*, and n is the number of observations.



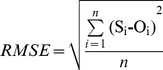
(8)E and R^2^ approaching one, and a RMSE near zero were indicators of improved model performance. Following model calibration, and validation, Grain-WUE and Biomass-WUE were calculated using Eqs. 3 and 4.

## Result and Analysis

### AquaCrop Model Calibration and Validation Results

**Figure 2:**
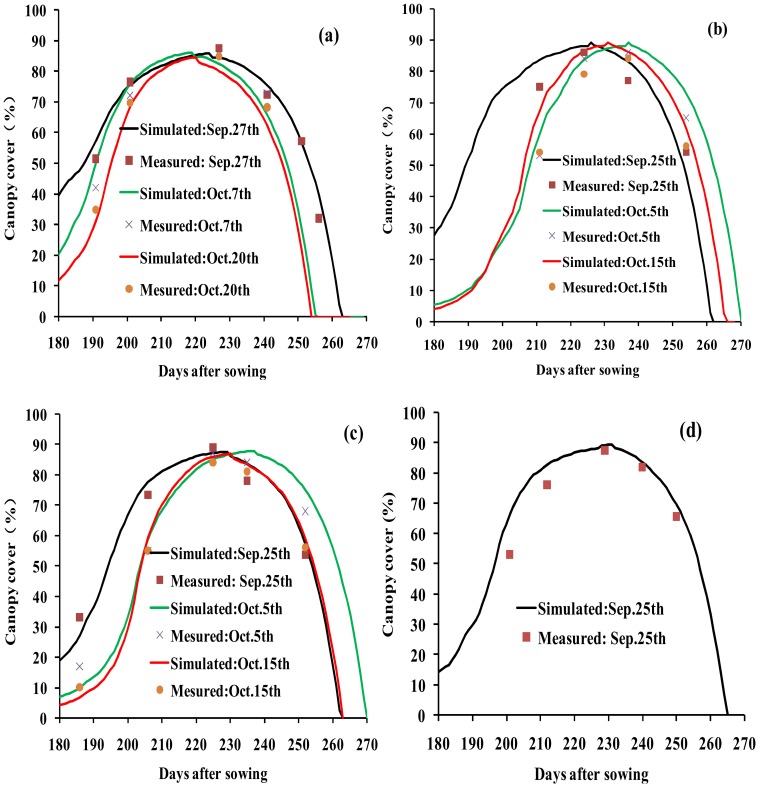
Simulated and measured canopy cover (CC) for winter wheat under different planting dates and irrigation strategies in the cropping season 2008/2009 (a), 2009/2010 (b), 2010/2011 (c) and 2011/2012 (d).

**Figure 3:**
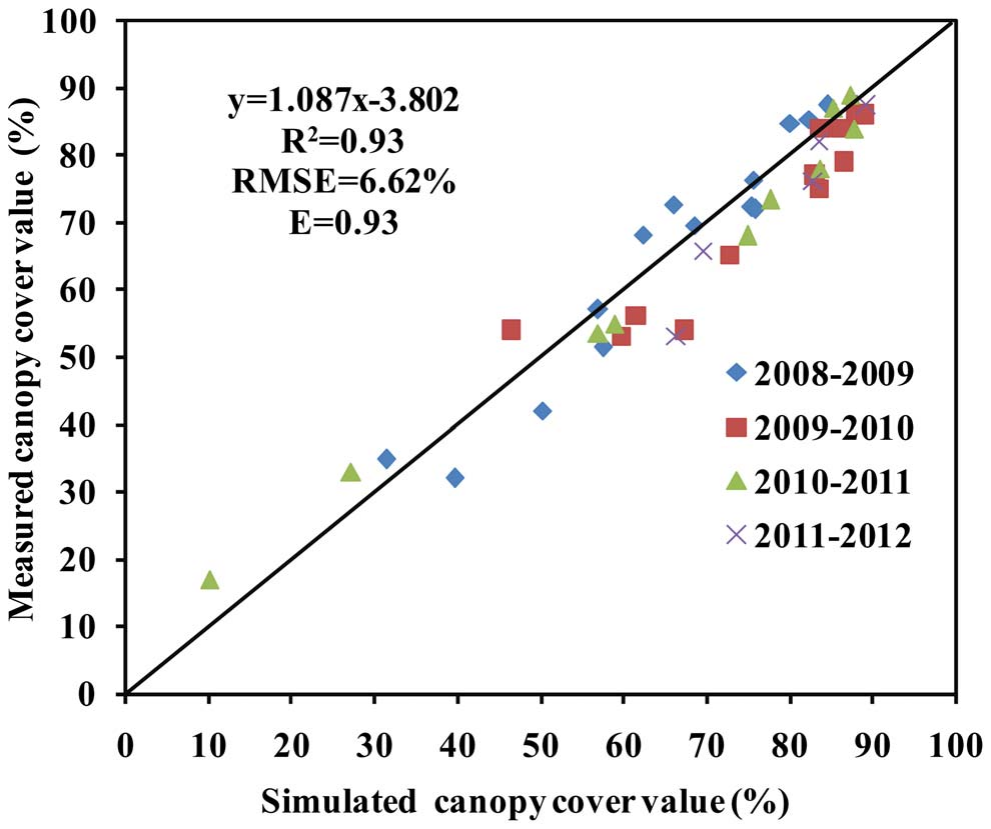
Relationship between the measured and simulated canopy cover (CC) in winter across 4 years. Note: x represents the simulated CC, y represents the measured CC. The intercept represents the relative error between the simulated CC and the measured CC. The slope represents the consistency between the simulated CC and the measured CC.

The crop parameters used to calibrate the AquaCrop model are presented in Table 4. Key stress parameters (e.g. canopy growth, canopy senescence stress coefficient) (P_upper_) were adjusted as needed to simulate CC. There was a strong linear relationship between the simulated and the measured CC (R^2^ = 0.93, RMSE = 6.62% and E = 0.93) for winter wheat under different planting dates, and irrigation strategies in the cropping season 2008/2009, 2009/2010, 2010/2011 and 2011/2012 (Figs. 2a, 2b, 2c, 2d and 3). The R^2^, RMSE and E of the simulated and measured CC were 0.91, 6.62% and 0.91, respectively in 2008/2009. And the R^2^, RMSE and E values of CC were 0.93, 4.94% and 0.93 in 2009/2010, 0.96, 7.19%, 0.94 in 2010/2011, and 0.96, 7.15%, and 0.93 in 2011/2012, respectively (Table 5).

**Table 4:** Input data of crop parameters used in AquaCrop model.

Description	Value	Unit
Base temperature	0	°C
Upper temperature	26	°C
Canopy growth coefficient (CGC): Increase in CC per day	0.03	%/day
Canopy decline coefficient (CDC): Decrease in CC per day at senescence	0.09	%/day
Maximum canopy cover (CC_x_)	90	%
Water productivity (NCWP)	15	g/cm^2^
Reference harvest index (HI)	46	%
Upper threshold for canopy expansion (P_upper_)	0.20	% of TAW
Lower threshold for canopy expansion (P_lower_)	0.65	% of TAW
Leaf expansion stress coefficient curve shape	3.0	–
Upper threshold for stomatal closure (P_upper_)	0.65	% of TAW
Minimum effective rooting depth	0.3	m
Maximum effective rooting depth	1.2	m
Canopy senescence stress coefficient (P_upper_)	0.70	% of TAW
Shape factor describing root zone expansion	1.5	–
Crop coefficient when canopy is complete but prior to senescence	1.1	–
Senescence stress coefficient curve shape	3.0	–
Allowable maximum increase of specified HI	15	%
Minimum air temperature below which pollination starts to fail	5	°C
Maximum air temperature above which pollination starts to fail	35	°C
Water Productivity normalized for ET_o_ and CO_2_ during yield formation	100	%
Time from sowing to emergence	7	Days
Time from sowing to flowering	232	Days
Time from sowing to start senescence	236	Days
Length of the flowering stage (days)	10	Days

**Table 5:** Simulated and measured canopy cover (CC) for winter wheat under different planting dates in 2008/2009, 2009/2010, 2010/2011 and 2011/2012.

Year	Plantingdate	Slope	Intercept	R^2^	RMSE(%)	E
2008–2009	28/9/2008	1.036	1.575	0.89	4.39	0.90
2008–2009	7/10/2008	1.251	−19.64	0.97	5.84	0.94
2008–2009	20/10/2008	1.018	2.032	0.98	4.25	0.96
2008–2009		1.060	−2.093	0.91	6.62	0.91
2009–2010	25/9/2009	1.201	−11.98	0.91	5.93	0.90
2009–2010	5/10/2009	1.082	−6.316	0.95	4.35	0.94
2009–2010	15/10/2009	0.930	8.443	0.92	7.02	0.91
2009–2010		1.120	−8.039	0.93	4.94	0.93
2010–2011^a^	25/9/2010	1.157	−12.31	0.97	5.73	0.94
2010–2011^a^	5/10/2010	1.180	−13.21	0.92	3.18	0.92
2010–2011^a^	15/10/2010	1.183	−7.109	0.96	3.78	0.95
2010–2011^a^		1.134	−7.996	0.96	7.19	0.94
2011–2012^a^	25/9/2011	0.743	24.35	0.96	7.15	0.93

Note: ^a^Validation data set: R^2^, determination coefficient; E, model efficiency; RMSE, root mean square of error.

The intercept represents the relative error between the simulated CC and the measured CC, The slope represents the consistency between the simulated CC and the measured CC.

**Figure 4:**
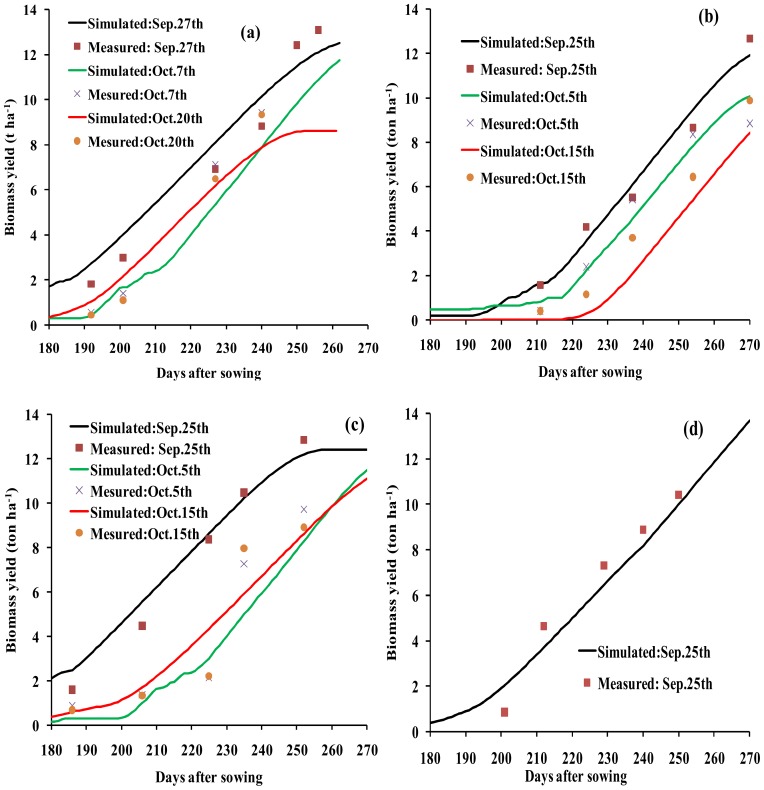
The simulated as compared with the measured aboveground biomass accumulation at different growth stages for winter wheat with different planting dates and irrigation strategies in the cropping season 2008/2009 (a), 2009/2010 (b), 2010/2011 (c) and 201/2012 (d).

**Figure 5:**
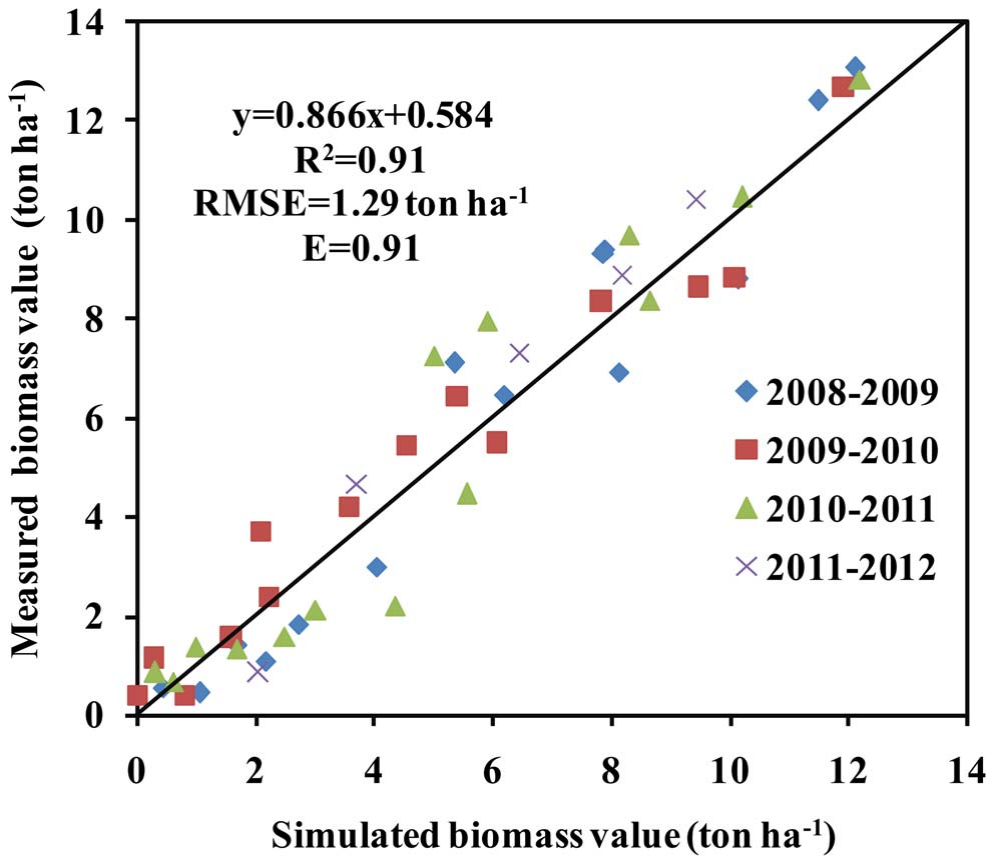
Relationship between the measured and simulated biomass yield (BY) in winter wheat across 4 years. Note: x represents the simulated BY, y represents the measured BY. The intercept represents the relative error between the simulated biomass yield and the measured BY. The slope represents the consistency between the simulated BY and the measured BY.

**Figure 6:**
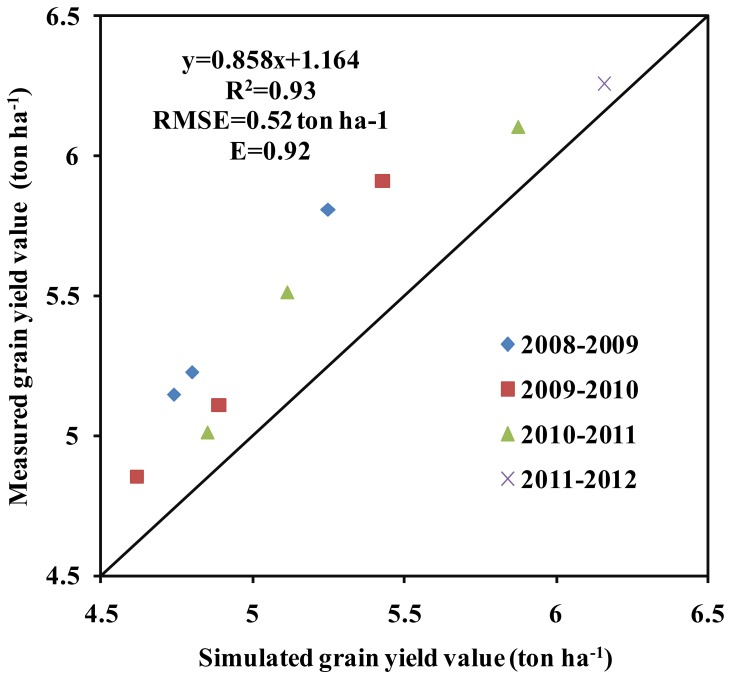
Relationship between the measured and simulated grain yield (GY) in winter wheat across 4 years. Note: x represents the simulated GY, y represents the measured GY. The intercept represents the relative error between the simulated GY and the measured GY. The slope represents the consistency between the simulated GY and the measured GY.

The simulated aboveground BY was similar to that measured (Figs. 4a, 4b, 4c and 4d). The stress coefficients were also adjusted, and readjusted as needed to simulated aboveground biomass. There was a strong relationship between measured and simulated BY across the four years (Fig. 5 and Table 6). The GY was also similar to the measured GY across all four years (R^2^, RMSE and E values of 0.93, 0.52 ton ha^−1^ and 0.92, respectively) (Fig. 6).

**Table 6:** Simulated and measured biomass yield (BY) for winter wheat under different planting dates in 2008/2009, 2009/2010, 2010/2011 and 2011–2012.

Year	Plantingdate	Slope	Intercept	R^2^	RMSE(ton ha^−1^)	E
2008–2009	28/9/2008	0.772	1.286	0.98	1.34	0.95
2008–2009	7/10/2008	0.787	−0.270	0.98	1.54	0.96
2008–2009	20/10/2008	0.802	−0.094	0.98	1.12	0.96
2008–2009		0.778	0.361	0.94	1.21	0.93
2009–2010	25/9/2009	0.993	1.315	0.92	1.39	0.92
2009–2010	5/10/2009	0.960	1.163	0.95	1.22	0.94
2009–2010	15/10/2009	0.954	1.331	0.97	1.27	0.95
2009–2010		0.945	1.284	0.95	1.29	0.94
2010–2011^a^	25/9/2010	0.809	0.617	0.97	1.21	0.95
2010–2011^a^	5/10/2010	1.043	0.378	0.97	1.37	0.95
2010–2011^a^	15/10/2010	0.797	0.019	0.97	1.84	0.94
2010–2011^a^		0.820	0.162	0.97	1.25	0.96
2011–2012^a^	25/9/2011	0.953	0.158	0.96	0.93	0.95

Note: ^a^Validation data set: R^2^, determination coefficient; E, model efficiency; RMSE, root mean square of error.

The intercept represents the relative error between the simulated BY and the measured BY, The slope represents the consistency between the simulated BY and the measured BY.

The R^2^, RMSE and E also showed good performance between the simulated and the measured values for CC (R^2^ = 0.89–0.98, RMSE = 3.18–7.19% and E = 0.90–0.96) and BY (R^2^ = 0.92–0.98, RMSE = 1.12–1.84 ton ha^−1^ and E = 0.92–0.96) (Tables 5 and 6). Higher R^2^ and E values and lower RMSE values indicated good model performance. The calibrated results were also consistent with the validated results for CC, BY and GY (Tables 5 and 6). These results suggest that the AquaCrop model is useful for simulating winter wheat CC, BY and GY under different planting dates, and irrigation strategies.

### Biomass, Grain Yield and Water Use Efficiency

**Figure 7:**
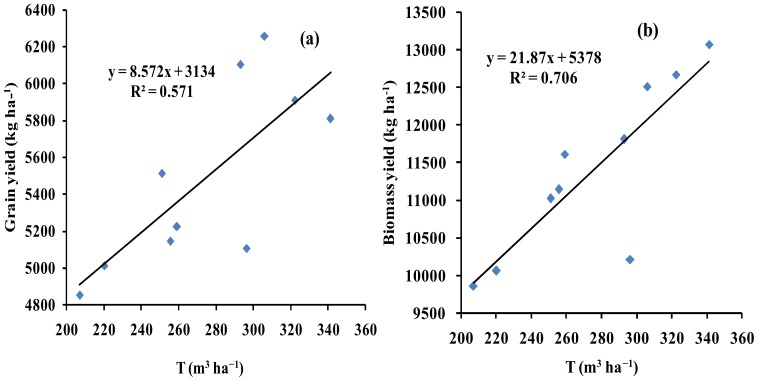
Relationships between the measured grain yield (GY), biomass yield (BY) and transpiration (T) in winter wheat. Note: (a) x represents transpiration, y represents the measured GY. The intercept represents the relative estimation error between transpiration and the measured GY. The slope represents the estimation consistency between transpiration and the measured GY. (b) x represents transpiration, y represents the measured BY. The intercept represents the relative estimation error between transpiration and the measured BY. The slope represents the estimation consistency between transpiration and the measured BY.

Winter wheat was planted on Sep. 25th (normal sowing), Sep. 28th (normal sowing), Oct. 5th (late sowing), Oct. 7th (late sowing), Oct. 15th (late sowing) and Oct. 20th (late sowing) during 2008/2011. Winter wheat that was planted on Sep. 25th and 28th had greater biomass and grain yield than did those planted on Oct. 5th, 7th, 15th, and 20th (Table 6). There was relatively more transpiration and biomass yield, yet lower grain yield in 2008 than in 2010, but there was relatively more transpiration (T), biomass yield, and even grain yield in 2008 than in 2009 (Table 7). The highest biomass and grain yield was obtained in crops planted on Sep. 28th 2008 and on Sep. 25th 2011, respectively; the lowest biomass and grain yield was obtained in crops planted on Oct. 15th 2009. A relatively higher grain and biomass yield per m^3^ of water was obtained from crops planted in 2010 than was in 2008 or 2009. Therefore, the biomass and grain yield water use efficiency (biomass-WUE and yield-WUE) was higher in 2010 than both 2008 and 2009. The biomass yield WUE was higher in 2008 than in 2009, yet the grain yield WUE was lower in 2008 than in 2009. Frequent irrigation with a small amount of water obviously improved grain yield in 2010/2011 and 2011/2012 and increased the biomass-WUE and grain-WUE (Table 5). A good relationship did exist between GY, BY, and T (R^2^ values of 0.57 and 0.71, respectively) (Fig. 7), thus suggesting that T might be used in estimating biomass and grain yield.

**Table 7:** Biomass-WUE and Grain-WUE in response to the seasonal transpiration over the different planting dates across years at Xiaotangshan experimental site.

Year	Planting date	Biomass (kg ha^−1^)	Grain (kg ha^−1^)	T (m^3^ ha^−1^)	Biomass-WUE (kg m^−3^)	Grain-WUE (kg m^−3^)
2008–2009	28/9/2008	13072	5808	341.2	3.83	1.70
2008–2009	7/10/2008	11612	5227	259.1	4.48	2.02
2008–2009	20/10/2008	11144	5146	255.7	4.36	2.01
2009–2010	25/9/2009	12666	5909	322.3	3.93	1.83
2009–2010	5/10/2009	10212	5106	296.3	3.45	1.72
2009–2010	15/10/2009	9861	4852	207	4.76	2.34
2010–2011	25/9/2010	11812	6103	292.9	4.03	2.08
2010–2011	5/10/2010	11024	5512	251.1	4.39	2.20
2010–2011	15/10/2010	10062	5013	220.2	4.57	2.28
2011–2012	25/9/2011	12514	6257	306	4.09	2.04

Note: T, transpiration; WUE, water use efficiency.

## Discussion

In this study, the AquaCrop model successfully predicted CC, BY GY in winter wheat. The crop parameters are adjusted to simulate CC, BY and GY for winter wheat under different planting dates and irrigation strategies. These adjustments were made to obtain more stable and closer relationships between the simulated values and the measured values. The results showed that the model calibration data sets from 2008/2009 and 2009/2010 were very consistent with the model validation data sets from 2010/2011 and 2011/2012. Good relationships were obtained between the simulated CC, BY and GY, and the measured CC, BY and GY across four years (Figs. 2,3,4,5 and 6, Tables 5 and 6). The best fitting model was obtained in 2009/2010, and the poorest fitting model was obtained in 2011/2012. These differences are very likely due to differences in rainfall and irrigation application (Fig. 1). Compared to 2008/2009, 2009/2010 and 2010/2011, the severe drought and inadequate irrigation occurred in 2011/2012. In the same year, CC, BY and GY were significantly different under different planting dates, likely due to accumulated temperature difference (Figs 3 and 4, Table 7). The results indicate that the AquaCrop model can be used to simulate CC, BY and GY for winter wheat under different planting dates and irrigation strategies. Our results (along with those in the Salemi et al. [39]), suggested that climate conditions, variety planted, and irrigation strategy could induce some differences in model simulations under different years. Heng et al. [34] demonstrated that the AquaCrop model is a good predictor of biomass and yield when irrigation is adequate; and this was corroborated by the results of this present study. In addition, the simulated BY was also consistent with the measured BY under different planting dates (Table 6 and Figs.4a–d). The CC results in this study were also similar to that observed in Salemi et al. [39], and Du et al. [40]. The results suggested that AquaCrop model could be used to simulate winter wheat CC, BY and GY under different planting dates and irrigation strategies. Winter wheat obtained higher biomass and grain yield when planted on Sep. 25th and Sep. 28th than on Oct. 5th, Oct. 7th, Oct. 15th and Oct. 20th (Table 7). This was likely due to the higher growing degree days (accumulated temperature) promoting CC growth, and BY, GY accumulation at the earlier planting date. The degree of CC affects the rate of transpiration and consequently BY and GY accumulation [32]. Therefore, the relatively higher BY and GY required relatively more temperature accumulation.

Biomass, and grain yield WUE decreased with increasing transpiration amount for all four years. This is consistent with that presented in Farahani et al. [32], but is not with that presented in Hedge [58]. In this present study, grain yield WUE ranged from 1.70 to 2.34 kg m^−3^, reaching its maximum value on Oct. 15th 2009. Wang et al. [21] reported that grain yield WUE for winter wheat was between 0.7 and 1.3 kg m^−3^, and Li et al. [50] reported it to be between 0.93 and 1.51 kg m^−3^ in WUE. These results are; however, not consistent with Wang et al. [26], and Li et al. [50], in which grain yield WUE was reported to be much greater. It indicated that winter wheat varieties developed faster, resulting in greater yield, leading to improvement in WUE. However, our results are consistent with that found in Fang et al. [59], in which the grain yield WUE ranged from 1.71 to 2.21 kg m^−3^. The BY and GY in 2010/2011 and 2011/2012 were higher than in 2008/2009 and 2009/2010. This suggested that the frequent irrigation could be used to increase BY, GY, and promote biomass yield WUE and grain yield WUE for winter wheat at drought stages. This might be one of the reasons that the effects of drought on BY and GY were reduced by the frequent irrigation, thereby improving WUE.

This study demonstrated that the AquaCrop model could be used to evaluate different planting date and irrigation strategies for winter wheat in NCP. Many others have conducted AquaCrop model application studies under different crops and environment conditions [32], [35], [37], [38], [43], [49]. These results indicated that AquaCrop model is stable and usable for different crops and environmental conditions. It is therefore plausible to use the AquaCrop model to improve irrigation management strategies that would maximize grain and biomass yield It is important to note that this study was limited to winter wheat in Xiaotangshan experimental site, Beijing, China. A subsequent study is focused on validating this model under deficit irrigation, thereby expanding the extent of this model’s application in the future.

## Conclusion

This paper demonstrated that the AquaCrop model adequately simulated the CC, BY, and GY of winter wheat under different planting dates and irrigation strategies. The simulated CC agreed well with the measured CC across all 4 years. The R^2^, RMSE, E of CC winter wheat ranged from 0.89 to 0.98, 3.18% to 7.19% and 0.90 to 0.96, respectively. The measured and simulated BY were also closely related. The AquaCrop model calibrated the BY with the prediction error statistics of 0.92< R^2^<0.98, 1.12< RMSE <1.84 ton ha^−1^ and 0.92< E<0.96. The simulated GY was also consistent with the measured GY with the R^2^, RMSE and E values of 0.93, 0.52 ton ha^−1^ and 0.92, respectively. The results demonstrated that frequent irrigation obviously improved BY, GY, biomass WUE and grain WUE for winter wheat in 2010/2011. These results suggest that the AquaCrop model could be used to predict CC, BY and GY of winter wheat with a high degree of reliability under various planting dates and irrigation strategies situations in the North China Plain (NCP).

## References

[1] Zhang HC (2000) Thoughts on cultivation techniques for high quality of wheat in China and its processing. Jiangsu Agricultural Sciences. 5: 2–6 (in Chinese)..

[2] Molden D (2003) A water-productivity framework for understanding and action. In: Kijne, J.W., Barker, R., Molden, D. (Eds.), Water Productivity in Agriculture: Limits and Opportunities for Improvement. International Water Management Institute, Colombo, Sri Lanka, pp. 1–18.

[3] Zwart SJ, Bastiaanssen WGM (2004) Review of measured crop water productivity values for irrigated wheat, rice, cotton and maize. Agriculture Water Management. 69: 115–133.

[4] Anac MS, Ali Ul M, Tuzel IH, Anac D, Okur B, et al.. (1999) Optimum irrigation schedules for cotton under deficit irrigation conditions. In: Kirda, C.,Moutonnet, P., Hera, C., Nielsen, D.R. (Eds.), Crop Yield Response to Deficit Irrigation. Kluwer Academic Publishers, Dordrecht, Boston, London, pp. 196–212.

[5] Raes D, Geerts S, Kipkorir E, Wellens J, Sahli A (2006) Simulation of yield decline as a result of water stress with a robust soil water balance model. Agriculture Water Management. 81: 335–357.

[6] Hill RW, Allen RG (1996) Simple irrigation calendars: a foundation for water management. In: Food and Agricultural Organization of the United Nations (FAO) (Ed.), Irrigation Scheduling: From Theory to Practice. Rome, Italy, pp. 69–74.

[7] Raes D, Sahli A, Van Looij J, Ben Mechlia N, Persoons E (2000) Charts for guiding irrigation in real time. Irrigation Drainage System 14: 343–352.

[8] De Nys E, Kipkorir EC, Sahli A, Vaes R, Raes D (2001) Design of farmer oriented irrigation charts by using a soil-water balance model. In: Proceedings of the 4th Inter Regional Conference on Environment-Water, ICID, pp. 235–243.

[9] Legesse A (2006) Sustainable irrigation in the Tigray Highlands of Northern Ethiopia. Master dissertation of the Master in Water Resources engineering. K.U. Leuven University, Leuven, Belgium.

[10] Farre F, Faci JM (2009) Deficit irrigation in maize for reducing agricultural water use in a Mediterranean environment. Agriculture Water Management. 96: 384–394.

[11] Kipkorir EC (2002) Optimal planning of deficit irrigation for multiple crop systems according to user specified strategy. Dissertations de Agricultura No. 514. Faculty of Agricultural Sciences. K.U. Leuven University, Belgium.

[12] Debaek P, Aboudrare A (2004) Adaptations of crop manage to water-limited environments. European Journal of Agronomy. 21: 433–446.

[13] Ali MH, Talukder MSU (2008) Increasing water productivity in crop production. A synthesis. Agricultural Water Management. 95: 1201–1213.

[14] Behera SK, Panda RK (2009) Integrated management of irrigation water and fertilizers for wheat crop using field experiments and simulation modeling. Agricultural Water Management. 96, 1532–1540.

[15] Blum FA (2009) Effective use of water (EUW) and not water-use efficiency (WUE) is the target of crop yield improvement under drought stress. Field Crops Research. 112: 119–123.

[16] Geerts S, Raes D, Gracia M, Miranda R, Cusicanqui JA, et al (2009) Simulating yield response of Quinoa to water availability with AquaCrop. Agronomy Journal. 101: 499–508.

[17] Jensen ME (1968) Water consumption by agricultural plants. In: Academic Press, New York Water Deficits in Plant Growth (T T Koslowski, ed. ). 1: 1–22.

[18] Rao NH, Sarma PBS, Chander S (1988) Irrigation scheduling under limited water supply. Agriculture Water Management. 15: 165–175.

[19] Rao NH, Sarma PBS, Chander S (1992) Real time adaptive irigation scheduling under a limited water supply. Agriculture Water Management. 20: 267–279.

[20] Penning de Vries FWT, Jansen DM, Berge ten HFM, Bakema A (1989) Simulation of Ecophysiological process of growth in several annual crops. Simulation Monographys, Pudoc, Wagenningen, pp. 82–96.

[21] De Wit CT (1970) Dynamic concepts in biology. In: Prediction and Management of Photosynthetic Productivity. Proceedings International Biological Program/Plant Production Technical Meeting. Wageningen, Netherlands: PUDOC, pp. 17–23.

[22] Downey LA (1972) Water-yield relations for non-forage crops. Journal of Irrigation and Drainage Division, American Society of Civil Engineers. 98: 107–115.

[23] Minhas BS, Parikh KS, Srinivasan TN (1974) Towards the structure of a production function for wheat yields with dated in Puts of irrigation water. Water Resource Research. 10: 383–393.

[24] Blank H (1975) Optimal irrigation decisions with limited water (D). Ph. D. Thesis, Department of Civil Engineering, Colorado State University, Fort Collins, CO.

[25] Stewart JI, Hagan RM, Pruitt WO (1976) Production function sand predicted irrigation programs for Principal crops as required for water resources planning and increased water use efficiency (R). Final Report, U.S. Department of Interior, Washington DC, 1976, pp. 80.

[26] Wang JP, Sun XF (2001) A Combining Water-Crop Relation Model. Irrigation and Drainage. 2: 76–78.

[27] Kang SZ, Cai HJ, Feng SY (2004) Technique innovation and research fields of modern agricultural and ecological water-saving in the future. Transactions of the Chinese Society of Agricultural Engineering. 20: 1–6 (in Chinese)..

[28] Doorenbos J, Kassam AH (1979) Yield response to water. FAO irrigation and drainage paper no. 33. FAO, Rome.

[29] Steduto P, Hsiao TC, Raes D, Fereres E (2009) AquaCrop-The FAO crop model to simulate yield response to water. I. Concepts. Agronomy Journal. 101: 426–437.

[30] Hsiao TC, Heng L, Steduto P, Roja-Lara B, Raes D, et al (2009) AquaCrop-The FAO model to simulate yield response to water: parametrization and testing for maize. Agronomy Journal. 101: 448–459.

[31] Raes D, Steduto P, Hsiao TC, Fereres E (2009a) AquaCrop-The FAO Crop Model to Simulate Yield Response to Water: Reference Manual Annexes., www.fao.org/nr/water/aquacrop.html.

[32] Farahani HJ, Izzi G, Oweis TY (2009) Parameterization and evaluation of the AquaCrop model for full and deficit irrigated cotton. Agronomy journal 101: 469–476.

[33] Garcia-Vila M, Fereres E, Mateos L, Orgaz F, Steduto P (2009) Deficit irrigation optimization of cotton with AquaCrop. Agronomy journal. 101: 477–487.

[34] Heng LK, Hsiao TC, Evett S, Howell T, Steduto P (2009) Validating the FAO AquaCrop model for irrigated and water deficient field maize. Agronomy Journal 101: 488–498.

[35] Vanuytrecht E, Raes D, Willems P (2011) Considering sink strength to model crop production under elevated atmospheric CO_2_ . Agricultural and Forest Meteorology 15: 1753–1762.

[36] Zinyengere N, Mhizha T, Mashonjowa E, Chipindu B, Geerts S, et al (2011) Using seasonal climate forecasts to improve maize production decision support in Zimbabwe. Agricultural and Forest Meteorology. 151: 1792–1799.

[37] Stricevic R, Cosic M, Djurovic N, Pejic B, Maksimovic L (2011) Assessment of the FAO AquaCrop model in the simulation of rainfed and supplementally irrigated maize, sugar beet and sunflower. Agriculture Water Management. 98: 1615–1621.

[38] Abedinpour M, Sarangi A, Rajput TBS, Singh M, Pathak H, et al (2012) Performance evaluation of AquaCrop model for maize crop in a semi-arid environment. Agricultural Water Management. 110: 55–66.

[39] Salemi H, Mohd-Soom MA, Lee TS, Mousavi SF, Ganji A, et al (2011) Application of AquaCrop model in deficit irrigation management of Winter wheat in arid region. African Journal of Agricultural Research. 610: 2204–2215.

[40] Du WY, He XK, Shamaila Z, Hu ZF, Zeng AJ, et al (2011) Yield and biomass prediction testing of aquaCrop model for winter wheat. Transactions of the Chinese Society for Agricultural Machinery.42: 174–183 (in Chinese)..

[41] Wang XX, Wang QJ, Fan J, Fu QP (2013) Evaluation of the AquaCrop model for simulating the impact of water deficits and different irrigation regimes on the biomass and yield of winter wheat grown on China’s Loess Plateau. Agricultural Water Management 129: 95–104.

[42] Soddu A, Deidda R, Marrocu M, Meloni R, Paniconi C, et al (2013) Climate variability and durum wheat adaptation using the AquaCrop model in southern Sardinia. Procedia Environmental Sciences 19: 830–835.

[43] Todorovic M, Albrizio R, Zivotic L, Abi saab M, Stwckle C, et al (2009) Assessment of AquaCrop, CropSyst, and WOFOST models in the simulation of sunflower growth under different water regimes. Agronomy Journal. 101: 509–521.

[44] Karunaratne AS, Azam-ali SN, Izzi G, Steduto P (2011) Calibraion and validation of FAO-AquaCrop model for irrigated and water deficient bambara groundnut. Experimental Agriculture 47: 509–527.

[45] Garcia-Vila M, Fereres E (2012) Combining the simulation crop model AquaCrop with an economic model for the optimization of irrigation management at farm level. European Journal Agronomy. 36: 21–31.

[46] Yuan M, Zhang L, Gou F, Su Z, Spiertz JHJ, et al (2013) Assessment of crop growth and water productivity for five C_3_ species in semi-arid Inner Mongolia. Agricultural Water Management 122: 28–38.

[47] Araya A, Keesstra SD, Stroosnijder L (2010a) Simulating yield response to water of Teff (Eragrostis tef) with FAO’s AquaCrop model. Field Crops Research. 116: 196–204.

[48] Araya A, Habtu S, Hadgu KM, Kebede A, Dejene T (2010b) Test of AquaCrop model in simulating biomass and yield of water deficient and irrigated barley (Hordeum vulgare). Agricultural Water Management. 97: 1838–1846.

[49] Abrha B, Delbecque N, Raes D, Tsegay A, Todorvovic M, et al (2012) Sowing strategies for barley (Hordeum vulgare L.) based on modeled yield response to water with AquaCrop. Experimental Agriculture. 48: 252–271.

[50] Li ZZ, Xu Y, Lu XJ, Hu KL, Jiang LH, et al (2011) Evaluation of the AquaCrop model for simulating biomass for Chinese green onion and soil water storage. Journal o f China Agricultural University.16: 59–66 (in Chinese)

[51] Katerji N, Campi P, Mastrorilli M (2013) Productivity, evapotranspiration, and water use efficiency of corn and tomato crops simulated by AquaCrop under contrasting water stressconditions in the Mediterranean region. Agricultural Water Management 130: 14–26.

[52] Allen RG, Periera LS, Raes D, Smith M (1998) Crop evapotranspiration. Guide-lines for computing crop water requirement. FAO Irrigation and Drainage Paper No.56. FAO, Rome.

[53] FAO (2009) ETo calculator version 3.1. In: Evapotranspiration from reference surface, FAO, Land and Water Division, Rome, Italy.

[54] Kar G, Verma HN, Singh R (2006) Effect of winter crop and supplementary irrigation on crop yield, water us efficiency and profitability in rainfed rice based on cropping system of eastern India. Agriculture Water Management. 79: 280–292.

[55] Oweis T, Hachum A (2006) Water harvesting and supplementary irrigation for improved water productivity of dry farming systems in West Asia and North Africa. Agriculture Water Management. 80: 57–73.

[56] Raes D, Steduto P, Hsiao TC, Fereres E (2009b) Crop Water Productivity. Calculation Procedures and Calibration Guidance. AquaCrop version 3.0. FAO, Land and Water Development Division, Rome.

[57] Raer D, Steduto P, Hsiao TC, Fereres E (2010) Reference Manual. Annexes-AquaCrop, Crop parameters. Annex I Section I.10 Wheat, A39–A42. FAO, Land and Water Development Division, Rome.

[58] Hedge DM (1987) The effect of soil water potential, method of irrigation, canopy temperature, yield and water use of radish. Horticultural Science. 62: 507–511.

[59] Fang QX, Chen YH, Li QQ, Yu SZ, Luo Y, et al (2004) Effect of irrigation on water use efficiency of winter wheat. Transactions of the Chinese Society of Agricultural Engineering. 20: 34–39 (in Chinese)..

